# The role of writing motives in the interplay between implicit theories, achievement goals, self-efficacy, and writing performance

**DOI:** 10.3389/fpsyg.2023.1149923

**Published:** 2023-04-17

**Authors:** Fien De Smedt, Yana Landrieu, Bram De Wever, Hilde Van Keer

**Affiliations:** Department of Educational Studies, Faculty of Psychology and Educational Sciences, Ghent University, Ghent, Belgium

**Keywords:** writing performance, implicit theories, achievement goals, self-efficacy, writing motives

## Abstract

It is well established that students’ motivation for writing is a key predictor of their writing performance. The aim of the current study is to study and map the relations underlying different motivational constructs (i.e., implicit theories, achievement goals, self-efficacy, and writing motives) and to investigate how these contribute to students’ writing performance. For that, 390 Flemish students in stage three of the academic track of secondary education (16–18  years old) completed questionnaires measuring their implicit theories of writing, achievement goals, self-efficacy for writing, and writing motives. Furthermore, they completed an argumentative writing test. Path analysis revealed statistically significant direct paths from (1) entity beliefs of writing to performance avoidance goals (*β* = 0.23), (2) mastery goals to self-efficacy for writing (*β*_argumentation_ = 0.14, *β*_regulation_ = 0.25, *β*_conventions_ = 0.18), performance-approach goals to self-efficacy for writing (*β*_argumentation_ = 0.38, *β*_regulation_ = 0.21, *β*_conventions_ = 0.25), and performance-avoidance goals to self-efficacy for writing (*β*_argumentation_ = −0.30, *β*_regulation_ = −0.24, *β*_conventions_ = −0.28), (3) self-efficacy for regulation to both autonomous (*β* = 0.20) and controlled motivation (*β* = −0.15), (4) mastery goals to autonomous motivation (*β* = 0.58), (5) performance approach and avoidance goals to controlled motivation (*β* = 0.18; *β* = 0.35), and (6) autonomous motivation to writing performance (*β* = 0.11). This study moves the field of writing motivation research forward by studying the contribution of implicit theories, achievement goals, and self-efficacy to students’ writing performance, via writing motives.

## 1. Introduction

To become proficient writers, students need to learn to skillfully manage production processes (e.g., idea generation and translating ideas into text), apply control mechanisms (e.g., monitoring the writing process), and rely on their long-term memory resources to retrieve, for instance, content and writing knowledge (Graham, 2018a,b). A great deal of effort and engagement is required in learning to manage such a complex skill as writing. To become a good writer and master this challenging skill, motivation is crucial for both initiating and sustaining persistence (Graham, 2018a,b; Camacho et al., 2021a). Writing motivation has been studied from various theoretical frameworks, leading to different but interrelated motivational concepts (Camacho et al., 2021a). The current study derives from self-theories (ST; Dweck, 1999), achievement goal theory (AGV; Elliot and Harackiewicz, 1996; Elliot and Church, 1997), self-efficacy theory (SET; Bandura, 1997), and self-determination theory (SDT; Ryan and Deci, 2000b, 2020) to study students’ implicit theories of writing, writing achievement goals, self-efficacy for writing, and writing motives, respectively. More particularly, we aim to disentangle how these different motivational concepts are related and how this complex interplay between motivational constructs contribute to students’ writing performance. In what follows, we conceptualize the motivational concepts central in this study and present the hypothesized relational model linking these concepts with each other and with writing performance.

## 2. Theoretical and empirical background

### 2.1. Implicit theories of writing

Based on self-theories (ST; Dweck, 1999), implicit theories pertain to students’ beliefs regarding a particular skill, such as reading, writing, or learning, and whether it is innate and unchangeable (i.e., entity theories or entity beliefs) or can be acquired or developed through dedication and hard work over time (i.e., growth theories or growth beliefs). Within the empirical writing research field, implicit theories of writing are considered an understudied motivational concept (Camacho et al., 2021a). Nevertheless, the few studies available provide evidence on the relation between implicit theories of writing and students’ writing performance by revealing that students with incremental beliefs of writing perform better in writing (Limpo and Alves, 2017; Camacho et al., 2022). Interestingly, these studies showed that implicit theories were not only directly related to writing performance (Camacho et al., 2022), but also indirectly via achievement goals and self-efficacy for writing (Limpo and Alves, 2017). For instance, Limpo and Alves (2017) showed that entity theories of writing were negatively related to mastery goals, which, in turn, contributed positively to writing performance via self-efficacy for regulation.

### 2.2. Writing achievement goals

Writing is a goal-directed activity in which a (community of) writer(s) purposefully writes a text to a certain audience to achieve a certain goal (e.g., persuade, inform) (Graham, 2018a,b). According to achievement goals theorists (AGT), writers can have different reasons for pursuing specific writing goals. The trichotomous model of achievement goals, which is most widely applied in writing research, distinguishes mastery-oriented, performance-approach, and performance-avoidance goals (Elliot and Harackiewicz, 1996; Elliot and Church, 1997). Following this trichotomous model, mastery-oriented writers commit to writing for the sake of the act itself and to become skillful in mastering it. Performance-oriented writers are directed to maximize their perceived competence. Avoidance-oriented writers tend to avoid the appearance of incompetence in writing. Within the empirical writing research field, the relation between achievement goals and students’ writing performance has been studied across text genres and across educational levels (Camacho et al., 2021a). In general, writing research studies are rather consistent on the direct positive association between mastery-oriented goals and students’ writing performance on the one hand (Pajares and Cheong, 2003; Kaplan et al., 2009; Camacho et al., 2022), and the direct negative relation between performance-approach and avoidance goals and students’ writing performance on the other hand (Pajares and Cheong, 2003; Hamilton et al., 2013; Troia et al., 2013; Camacho et al., 2022). However, prior studies also showed that the role of writing achievement goals to predict students’ writing performance becomes more complex when other motivational variables are considered as well (e.g., self-efficacy for writing). For instance, Limpo and Alves (2017) and Soylu et al. (2017) showed that performance goals were indirectly related to writing performance via self-efficacy. However, the studies were not consistent in their findings. More particularly, Limpo and Alves (2017) found that mastery goals positively contributed to writing performance via self-efficacy for regulation. Contrarily, Soylu et al. (2017) did not find any direct or indirect relations between mastery goals and writing performance. However, they did find an indirect positive path between achievement-performance goals and writing performance and an indirect negative path of performance-avoidance goals and writing performance, both via self-efficacy for conventions. In sum, the indirect role of writing achievement goals in predicting students’ writing performance via other motivational concepts, such as self-efficacy, remains unclear.

### 2.3. Self-efficacy for writing

According to self-efficacy theory (SET; Bandura, 1997), self-efficacy beliefs pertain to one’s expectations of perceived capability. These self-efficacy beliefs impact how one will approach the task, the level of effort and persistence one brings to the task, and ultimately one’s actual performance. In the writing research field, students’ self-efficacy for writing is the most widely studied motivational construct and is considered as a key predictor of students’ writing performance (Camacho et al., 2021a). In this respect, the conceptualization of Bruning et al. (2013) is often used to study students’ self-efficacy for ideation (i.e., self-beliefs about the ability to generate ideas), conventions (i.e., self-beliefs about the ability to adhere to and apply writing rules), and regulation (i.e., self-beliefs about the ability to regulate behavior and emotions during writing). Prior writing research studies adopting this three-dimensional model to study the role of self-efficacy on students’ writing performance, resulted in mixed findings. More particularly, both Soylu et al. (2017) and Zumbrunn et al. (2020) found positive associations between self-efficacy for conventions and students’ scores on a statewide writing assessment and on students’ writing grades, respectively. Limpo and Alves (2017) and Camacho et al. (2021b), in turn, reported on positive relations between self-efficacy for regulation and students’ writing performance. Finally, De Smedt et al. (2016) did not find any evidence on the predictive role of self-efficacy for conventions or regulation on writing performance, but they did report a positive relation between self-efficacy for ideation and text quality. Furthermore, in a subsequent structural equation modeling study, De Smedt et al. (2018b) explored motivational and cognitive predictors of writing performance and results revealed no direct relation between self-efficacy for writing and writing performance when writing motives (i.e., autonomous and controlled writing motivation) were taken into account. In sum, the predictive role of different self-efficacy beliefs for writing (i.e., ideation, conventions, and regulation) on students’ writing performance remains unclear, especially when other motivational predictors, such as writing motives, are simultaneously studied.

### 2.4. Writing motives

Self-determination theory (SDT) is a theory of human motivation that has been developed through empirical research. It is particularly attractive to educational researchers due to its unique conceptualization of motivation, which redefines the traditional distinction between intrinsic and extrinsic motivation. More particularly, SDT conceptualizes subtypes of motivation with differing levels of regulation resulting in a continuum: amotivation (i.e., absence of motivation), external regulation (i.e., driven by external pressure), introjected regulation (i.e., driven by internal pressure), identified regulation (i.e., driven by values), and intrinsic regulation (i.e., driven by inherent fulfillment) (Ryan and Deci, 2000b, 2020). Based on SDT, writing researchers differentiated between autonomous and controlled motives for writing instead of intrinsic and extrinsic motives (De Smedt et al., 2020a,b, 2022). While autonomously motivated writers personally endorse the value of writing or inherently enjoy writing, controlled motivated writers are driven by externally or internally imposed rewards and punishments. In line with the core hypotheses of SDT, studies in the field of writing research showed that autonomously motivated students write texts of higher quality compared to texts produced by more controlled motivated students (De Smedt et al., 2016, 2018b; Rasteiro and Limpo, 2022). Despite this empirical evidence on the relations between writing motives and performance, research on writing motives and how these relate to other motivational writing constructs, is still scarce within the writing research field.

## 3. The relational model

**Figure 1:**
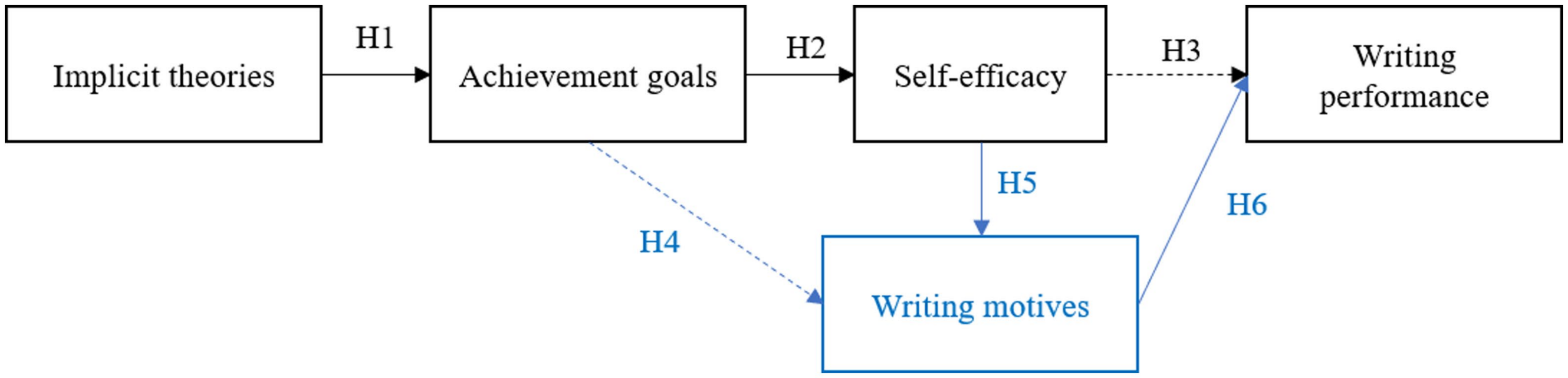
Hypothesized relational model. *H1*, *H2*, and *H3* were studied in Limpo and Alves (2017). The current study expands this by additionally including *H4*, *H5*, and *H6* in the relational model (in blue).

As outlined in the theoretical background, writing motivation has been studied from various theoretical frameworks, leading to different but interrelated motivational concepts (i.e., implicit theories of writing deriving from ST, writing achievement goals deriving from AGT, self-efficacy for writing deriving from SET, and writing motives deriving from SDT). In the current study, we aim to disentangle how these different motivational concepts are related and how these contribute directly or indirectly to students’ writing performance. This study builds on prior studies in which the relations between implicit theories of writing and achievement goals (e.g., Camacho et al., 2022) and the relations between achievement goals and self-efficacy for writing (Soylu et al., 2017) are studied in view of predicting students’ writing performance. In this respect, the study of Limpo and Alves (2017) is particularly inspiring as they studied how implicit theories relate to achievement goals (see Figure 1, *H1*), which, in turn, are associated with self-efficacy for writing (see Figure 1, *H2*), which ultimately relates to writing performance (see Figure 1, *H3*). The hypothesized relational model that was studied by Limpo and Alves (2017) is visualized in black in Figure 1. The results of this model are presented in detail in sections 3.1, 3.2, and 3.3. The current study expands the relational model of Limpo and Alves (2017) by including students’ writing motives in the hypothesized relational model (extensions in blue in Figure 1). There is increased attention in the writing research field to study writing motives as conceptualized by SDT (De Smedt et al., 2018a,b, 2020b, 2022). Despite the empirical evidence on the relations between writing motives and performance, research on writing motives and how these relate to other motivational writing constructs, is still limited. In the current study, we aim to understand the role of writing motives in the complex interplay of motivational predictors and students’ writing performance. We therefore also study the relations between achievement goals and writing motives (*H4*), self-efficacy and writing motives (*H5*), and writing motives and writing performance (*H6*). In sections 3.4, 3.5, and 3.6 we will present the hypothesized relations between writing motives and achievement goals, self-efficacy, and writing performance. Finally, in section 3.7, we will present assumed indirect paths in the hypothesized relational model based on prior research.

### 3.1. Hypothesis 1 (*H1*): Implicit theories are related to achievement goals

According ST (Dweck, 1999) in general and based on the studies of Camacho et al. (2022) and Limpo and Alves (2017) in particular, we hypothesize that implicit theories reflecting a fixed mindset are positively associated with performance-avoidance goals and negatively related to mastery goals.

### 3.2. Hypothesis 2 (*H2*): Achievement goals are related to self-efficacy beliefs

We anticipate that (a) mastery goals are positively associated with the three types of self-efficacy (i.e., ideation, conventions, regulation; Limpo and Alves, 2017), (b) performance-approach goals are positively related to all three dimensions of self-efficacy for writing (Soylu et al., 2017), and (c) performance-avoidance goals are negatively related to all three dimensions of self-efficacy beliefs for writing (Soylu et al., 2017).

### 3.3. Hypothesis 3 (*H3*): Self-efficacy beliefs are related to writing performance

Although self-efficacy for writing is considered a key predictor of writing performance (Camacho et al., 2021a), studies revealed mixed results on the predictive role of self-efficacy when conceptualized as a three-dimensional construct (i.e., ideation, conventions, and regulation) and when simultaneously other motivational constructs are considered (Limpo and Alves, 2017; Soylu et al., 2017). Based on De Smedt et al. (2018b) who did not find a direct relation between self-efficacy for writing and writing performance when taking into account writing motives, we hypothesize no direct association between self-efficacy for writing and writing performance.

### 3.4. Hypothesis 4 (*H4*): Achievement goals are related to writing motives

Deci and Ryan (2000) claimed that to understand the effect of achievement goals on human behavior, it is crucial to understand why people pursue them and thus to consider people’s motives. In this respect, Dweck (1985) theorized that when students are oriented toward mastery goals, the intrinsic motivation system is involved in initiating and sustaining the activity, while performance-approach or performance-avoidance goals can undermine intrinsic motivation. The alignment between achievement goals and extrinsic motivation is, however, not that straightforward given the full array of extrinsic motivations within SDT as presented in the theoretical background (Deci and Ryan, 2000). In line with prior writing research deriving from the SDT perspective (De Smedt et al., 2020b), we opted for including autonomous and controlled motivation (instead of intrinsic and various types of extrinsic motivation). We refrain from posing hypotheses on the relations between achievement goals and writing motives for two reasons. First, although motivational theorists have pointed out the alignment between AGT and SDT by studying the relations between achievement goals and extrinsic and intrinsic motives (Dweck, 1985; Elliot and Harackiewicz, 1996; Deci and Ryan, 2000), there is no empirical research to date relating the trichotomous model of achievement goals with autonomous and controlled motivation. Second, the current study is the first to introduce possible relations between achievement goals and writing motives within the writing research field.

### 3.5. Hypothesis 5 (*H5*): Self-efficacy beliefs are related to writing motives

In SDT, it is hypothesized that the fulfilled need for competence has a direct relation with motivation, indicating the association between self-efficacy beliefs and motives (Sweet et al., 2012; Kyndt et al., 2019). Based on prior research on students’ learning, indicating the positive relation between self-efficacy and autonomous motivation (Katz et al., 2014), we anticipate that students’ self-efficacy for writing is positively related to autonomous writing motives. We refrain from posing specific hypotheses on which types of self-efficacy (i.e., ideation, conventions, regulation) relate to which types of writing motives (i.e., autonomous, controlled) as no prior studies within the writing research field have studied the relation between self-efficacy beliefs and writing motives in such depth.

### 3.6. Hypothesis 6 (*H6*): Writing motives are related to writing performance

In line with the core hypotheses of SDT (Ryan and Deci, 2000b, 2020) and based on prior empirical writing research studies (De Smedt et al., 2016, 2018b; Rasteiro and Limpo, 2022), we expect autonomous writing motives to be positively related to writing performance, while controlled writing motives will be negatively associated with writing performance.

### 3.7. Hypothesized indirect paths

Next, to the abovementioned hypothesized direct paths, we also investigated possible indirect paths. Based on the model of Limpo and Alves (2017) we study the indirect paths between (1) implicit theories and self-efficacy, via achievement goals (*H1* + *H2*) and (2) achievement goals and writing performance, via self-efficacy (*H2* + *H3*). More particularly, we hypothesize that implicit theories reflecting a growth mindset will positively contribute to self-efficacy for conventions, argumentation, and regulation, via mastery goals (Limpo and Alves, 2017). Additionally, we anticipate that mastery goals will be related indirectly to writing performance, via self-efficacy for regulation (Limpo and Alves, 2017).

Given the novelty of including writing motives in the relational model, we refrain from posing specific hypotheses related to the indirect paths between (1) implicit theories and writing motives, via achievement goals (*H1* + *H4*), (2) achievement goals and writing motives, via self-efficacy (*H2* + *H5*), (3) achievement goals and writing performance, via writing motives (*H4* + *H6*), and (4) self-efficacy and writing performance, via writing motives (*H5* + *H6*).

## 4. Methodology

### 4.1. Participants

Secondary education in Flanders is aimed at students aged 12–18. The structure of secondary education comprises three stages (each consisting of 2 years). This study focusses on students who are enrolled in stage three of the academic track which prepares students to pass on to tertiary education. In total, 390 Flemish students in stage three of the academic track of secondary education participated (16–18 years old). The majority of the students identified themselves as female (62.3%), while 37.7% identified themselves as male. 85.1% of the participants were Dutch (the language of instruction) speaking, 7.7% spoke a foreign language at home, and 7.7% were bilingual (i.e., speaking Dutch and another foreign language at home). According to the attainment targets in Flanders, students in stage three of the academic track are expected to be able to write argumentative texts. However, instruction on how writing is taught and the time spent on writing in Flemish classes varies considerably (De Smedt et al., 2016; De Smedt and Van Keer, 2017). To gain insight into students’ experiences with argumentative texts, we explained the aim of an argumentative text and showed students a model text. Afterward, we asked students how many argumentative texts they have written during the past 6 months. Results showed large variation in students’ writing experience with the argumentative genre (35.1% did not write an argumentative text, 33.6% wrote one argumentative text, and 31.3% wrote more than one argumentative text in the past 6 months).

### 4.2. Data collection procedure

Data collection took place in the spring of 2021 by the second author. Given the COVID-19 pandemic, some schools were closed while others were open. Also, some classes were organized face-to-face, while other classes were online. Therefore, we opted for collecting all data digitally. First, students completed an informed consent. Given that the participating students were minors older than 16, the parents of the participating students were provided with a passive informed consent form. After students’ consent, they completed an online questionnaire measuring students’ implicit beliefs, achievement goals, self-efficacy for writing, and writing motives. Furthermore, they also completed an argumentative writing test. The questionnaires and the writing test were in Dutch, which is the language of instruction in Flanders and the first language of the majority of the participating students (85.1%). The questionnaires were completed online by the students either during class hours or at home using LimeSurvey GmbH (2012). The writing test was administered digitally during class hours when students were in class to ensure that students were not consulting any other sources than the provided source texts. More information on the questionnaires and writing test is provided in section 4.3.

### 4.3. Measures

#### 4.3.1. Implicit theories

The Implicit Theories of Writing Scale (ITW; Limpo and Alves, 2014, 2017) was administered to measure students’ beliefs about the malleability of their writing skills. Students need to complete 3 items on a six-point Likert scale indicating their level of agreement (e.g., No matter how many texts I write, their quality will always be the same). Higher scores on the scale indicate entity beliefs about writing (i.e., fixed mindset), while lower scores on the scale indicate incremental beliefs about writing (i.e., growth mindset). The structure and the fit of the ITW has been tested in prior studies with Portuguese students (Limpo and Alves, 2014, 2017; Camacho et al., 2022) but not yet with Flemish students. In the current study, we confirmed the stability of the one-factor model which provided a good fit to the data according CFI (YB *χ*^2^ (1) = 21.46, *p* < 0.001, CFI = 0.94, RMSEA = 0.26, SRMR = 0.24). High values of RMSEA and SRMR could be explained by the small number of degrees of freedom in the measurement model (Kenny et al., 2015). Finally, reliability analyses indicated a high internal consistency of the ITW scale (Bentler’s *ρ* = 0.87).

#### 4.3.2. Achievement goals

The Writing Achievement Goals Scale (WAGS; Soylu et al., 2017) was used to measure students’ goals or intentions when writing. The WAGS contains 12 items on a five-point Likert scale probing students’ mastery goals (i.e., the goal of the writer is to become a better writer), performance-approach goals (i.e., the goal of the writer is to maximize their perceived competence), and performance-avoidance goals (i.e., the goal of the writer is to avoid failure). The WAGS was tested in prior studies (Limpo and Alves, 2017; Soylu et al., 2017; Camacho et al., 2022), but has never been used with Flemish students. Therefore, we conducted confirmatory factor analyses to confirm the three-factor model. Results showed a good model fit (YB *χ*^2^ (50) = 156.27, *p* < 0.001, CFI = 0.93, RMSEA = 0.08, SRMR = 0.05). Furthermore, reliability analyses revealed that the three subscales were internally consistent (mastery goals: Bentler’s *ρ* = 0.82; performance-approach goals: Bentler’s *ρ* = 0.73; and performance-avoidance goals: Bentler’s *ρ* = 0.81).

#### 4.3.3. Self-efficacy for writing

The Self-Efficacy for Writing Scale (SEWS; Bruning et al., 2013) was administered to assess students’ self-efficacy for writing. The SEWS contains 16 statements which students have to complete by indicating their level of confidence ranging from 0 (no confidence) to 100 (complete confidence). The original SEWS consists of three subscales: self-efficacy for conventions (i.e., level of confidence to adhere to writing conventions such as correctly spelling words), self-efficacy for regulation (i.e., level of confidence to regulate the writing behavior and emotions, for instance by staying concentrated during the writing task), and self-efficacy for ideation (i.e., level of confidence to generate ideas for writing). In the current study, the subscale focused on ideation was slightly adapted to map students’ self-efficacy for argumentation (De Smedt et al., 2022). For instance, the original item “I can put my ideas into writing” was rephrased as “I can write my arguments into a text.” The structure and fit of the adjusted SEWS in the context of argumentative writing has previously been tested with Flemish students (De Smedt et al., 2022). In the current study, we confirmed this three-factor model (YB *χ*^2^ (101) = 307.23, *p* < 0.001, CFI = 0.91, RMSEA = 0.09, SRMR = 0.06) and reliability analyses showed that the three subscales were reliable (self-efficacy for conventions: Bentler’s *ρ* = 0.87, self-efficacy for regulation: Bentler’s *ρ* = 0.86, and self-efficacy for argumentation: Bentler’s *ρ* = 0.91).

#### 4.3.4. Writing motives

Students’ writing motives were measured using the SRQ-Writing Motivation (De Smedt et al., 2018b, 2020b). The SRQ-Writing Motivation consists of 18 items on a five-point Likert scale measuring students’ autonomous and controlled writing motivation. Autonomous motives for writing originate from students’ intrinsic interest in writing or from their appreciation for writing. Controlled motives for writing originate from external or internal feelings of pressure to write. The structure and fit of the SRQ-Writing Motivation was previously tested with Flemish secondary school students (De Smedt et al., 2020b, 2022) and the two-factor structure was confirmed in the current study as well (YB *χ*^2^ (136) = 3150.42, *p* < 0.001, CFI = 0.91, RMSEA = 0.09, SRMR = 0.09). Additionally, the internal consistencies of both subscales were acceptable to high (autonomous motivation: Bentler’s *ρ* = 0.92 and controlled motivation: Bentler’s *ρ* = 0.76).

#### 4.3.5. Writing performance

Students completed a previously developed integrated argumentative writing test based on two informational source texts (Landrieu et al., 2022). Students were instructed to take a stance in the discussion on lowering voting rights from 16 years old and to convince the readers of their position. They had 45 min to finish their argumentative text by including information from the source texts and additionally discussing their own opinion. Four trained raters assessed all texts holistically using a benchmark scale with five prototypical texts ranging from low quality to high quality. The selection of the five benchmark texts was based on the reliable rank order of argumentative texts on voting rights written by Flemish students in stage three of the academic track (Separation Scale Reliability = 0.83) presented in Landrieu et al. (2022). More particularly, we selected the benchmark texts with a standardized *z*-score of-2, −1, 0, 1, and 2 and placed the texts on a continuous scale in which the score of the benchmark with an average text quality was 100, and the interval between benchmarks was 15 (For more information on the procedure of selecting benchmark texts based on a rank order, see De Smedt et al., 2020a). This scale with five benchmark texts representing different text quality scores (*cf.*, scores 70, 85, 100, 115, and 130) supported the raters in holistically assessing the quality of the texts. In view of interrater reliability, 9.2% of the texts were double-scored revealing an Intraclass Correlation Coefficient (ICC) of.72.

### 4.4. Data analysis

The hypothesized relational model was evaluated with path analyses in the lavaan package in R (Rosseel, 2012; R Core Team, 2019). Because the data were not normally distributed (skewness values ranging from-1.07 to 0.25 and kurtosis values ranging from −0.66 to 2.99), we applied the robust maximum likelihood as method of estimation. To evaluate the model fit we used the YB-scaled chi-square statistic, the confirmatory fit index (CFI), the root-mean-square error of approximation (RMSEA), and the standardized root mean residual (SRMR). CFI values greater than.90, RMSEA values less.10, and SRMR equal or lower than 0.8 are considered adequate fits (Hu and Bentler, 1999).

## 5. Results

### 5.1. Descriptive results

Table 1 displays descriptive results and correlations between all study variables. Based on the significant positive correlations within the achievement goals on the one hand and the different types of self-efficacy for writing on the other hand, it was decided to include these associations in the path model. As the correlation between the writing motives was not statistically significant, no association between autonomous and controlled motives was included in the path model.

**Table 1:** Descriptive statistics and correlations for all study variables.

	(1)^a^	(2)^b^	(3)^b^	(4)^b^	(5)^c^	(6)^c^	(7)^c^	(8)^b^	(9)^b^	(10)^d^
*M* (*SD*)	2.97 (0.92)	3.46 (0.82)	2.63 (0.90)	2.52 (0.92)	65.81 (13.69)	62.43 (17.71)	77.24 (12.81)	20.87 (0.93)	3.00 (0.70)	84.40 (15.81)
(1) Implicit theories^a^	1									
(2) Mastery goals^b^	−0.04	1								
(3) Performance-approach goals^b^	0.11*	0.34**	1							
(4) Performance-avoidance goals^b^	0.24**	0.20**	0.46**	1						
(5) Self-efficacy for argumentation^c^	−0.20**	0.21**	0.29**	−0.10	1					
(6) Self-efficacy for regulation^c^	−0.23**	0.27**	0.18**	−0.10*	0.48**	1				
(7) Self-efficacy for conventions^c^	−0.16**	0.21**	0.18**	−0.13**	0.46**	0.46**	1			
(8) Autonomous writing motives^b^	−0.05	0.64**	0.28**	0.10	0.22**	0.35**	0.21**	1		
(9) Controlled writing motives^b^	0.22**	0.17**	0.33**	0.47**	−0.12*	−0.15**	−0.05	0.04	1	
(10) Writing performance^d^	0.06	0.09	−0.00	−0.05	−0.05	−0.05	−0.03	0.08	0.07	1

*p < 0.01; **p < 0.001.

a 6-Point Likert scale.

b 5-Point Likert scale.

c 100-Point scale.

d Benchmark text with score 100 represents an average text quality.

### 5.2. Path analysis results

**Figure 2:**
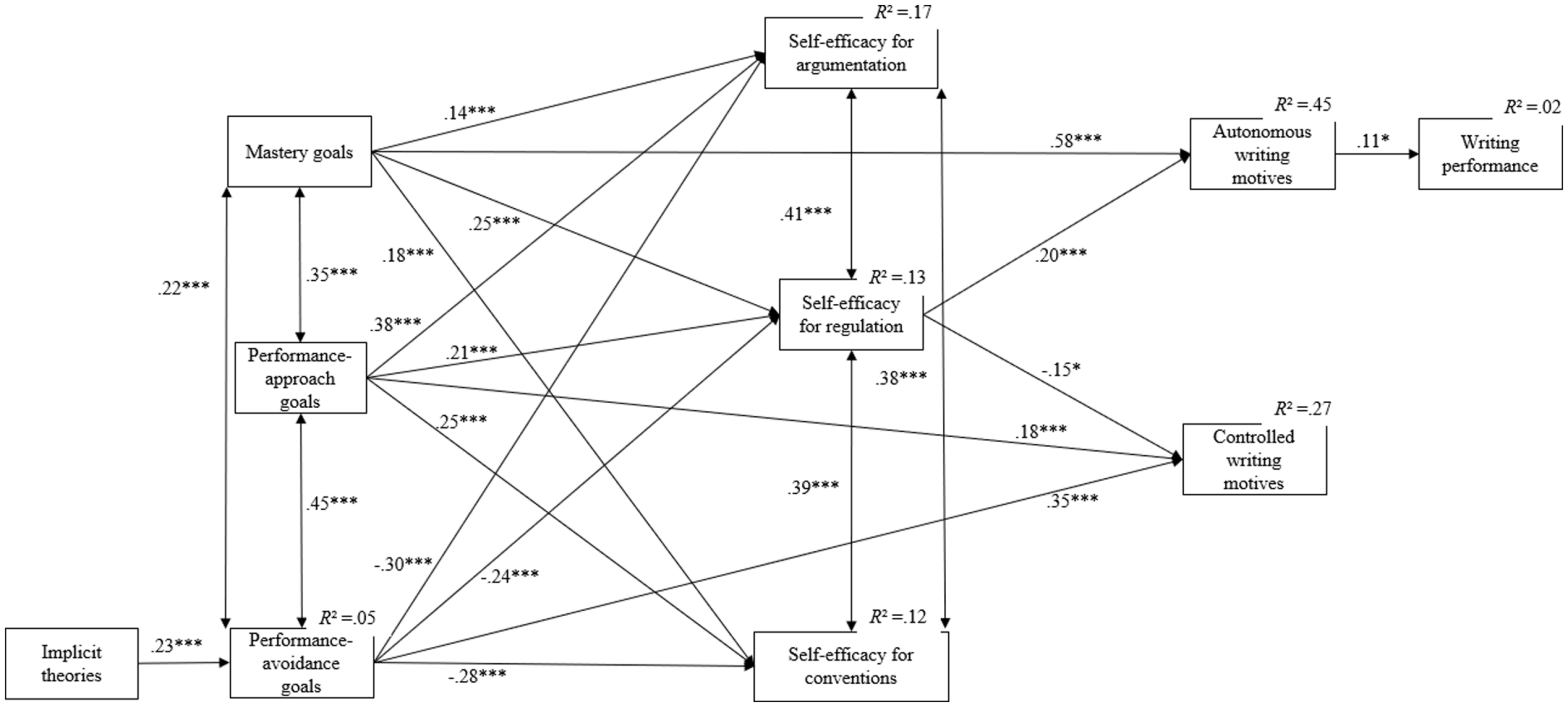
Significant standardized path coefficients of the path model (*p < 0.05; **p < 0.01; ***p < 0.001).

Results showed that the proposed model fitted the data well, *χ*^2^ (10) = 29.86, *p* < 0.001, CFI = 0.974, RMSEA = 0.075, SRMR = 0.043.^1^ Figure 2 presents the standardized betas for the statistically significant direct paths. The results for each of these paths will be presented in detail according the proposed hypotheses.

#### 5.2.1. Hypothesis 1: Implicit theories are related to achievement goals

As hypothesized, entity beliefs reflecting a fixed mindset are positively related to performance-avoidance goals (*β* = 0.23, *p* < 0.001). No relation was found between students’ entity beliefs and performance-approach goals (*β* = 0.10, *p* = 0.11). Contrary to our hypotheses, no relations were found between students’ implicit theories on writing and their mastery goals (*β* = −0.03, *p* = 0.65).

#### 5.2.2. Hypothesis 2: Achievement goals are related to self-efficacy beliefs

As predicted in the hypothesized relational model, students’ achievement goals are related to students’ self-efficacy beliefs for writing. More particularly, both mastery goals and performance-approach goals are positively associated with student’s self-efficacy for argumentation (*β* = 0.14, *p* < 0.001 and *β* = 0.38, *p* < 0.001, respectively), regulation (*β* = 0.25, *p* < 0.001 and *β* = 0.21, *p* < 0.001, respectively), and conventions (*β* = 0.18, *p* < 0.001 and *β* = 0.25, *p* < 0.001, respectively). On the contrary, but also in line with the hypothesized relational model, performance-avoidance goals were negatively related to student’s self-efficacy (argumentation: *β* = −0.30, *p* < 0.001; regulation: *β* = −0.24, *p* < 0.001; conventions: *β* = −0.28, *p* < 0.001).

#### 5.2.3. Hypothesis 3: Self-efficacy beliefs are related to writing performance

As expected in the proposed hypothesized relational model, the results showed no significant relations between self-efficacy beliefs and students’ writing performance (argumentation: *β* = −0.06, *p* = 0.31; regulation: *β* = −0.08, *p* = 0.15; and conventions *β* = 0.07, *p* = 0.23).

#### 5.2.4. Hypothesis 4: Achievement goals are related to writing motives

Path analytical results showed that students’ mastery goals were positively related to autonomous motives for writing (*β* = 0.58, *p* < 0.001), while both performance-approach goals (*β* = 0.18, *p* < 0.001) and performance-avoidance goals (*β* = 0.35, *p* < 0.001) were positively correlated with controlled motives for writing.

#### 5.2.5. Hypothesis 5: Self-efficacy beliefs are related to writing motives

Path analyses confirmed the hypothesis predicting the positive relation between self-efficacy beliefs and autonomous writing motives. More particularly, the results showed that self-efficacy for regulation was positively related to autonomous writing motivation (*β* = 0.20, *p* < 0.001). Although not hypothesized, the results also showed that self-efficacy for regulation was negatively associated with controlled writing motivation (*β* = −0.15, *p* = 0.012).

#### 5.2.6. Hypothesis 6: Writing motives are related to writing performance

In line with the proposed hypotheses, path analyses indicated a positive association between autonomous writing motives and students’ writing performance (*β* = 0.11, *p* = 0.047). Contrary to the predictions, no significant relationship was found between controlled writing motives and writing performance (*β* = 0.04, *p* = 0.43).

#### 5.2.7. Hypothesis 7: Indirect paths

As for the statistically significant indirect paths, the results indicated that entity beliefs were negatively related to self-efficacy for argumentation (*β* = −0.07, *p* = 0.006), regulation (*β* = −0.06, *p* = 0.011), and conventions (*β* = −0.07, *p* = 0.005), via performance-avoidance goals (*cf.*, *H1* + *H2*). Furthermore, the results showed that achievement goals contributed to writing motives via self-efficacy for regulation. More particularly, mastery goals (*β* = 0.05, *p* = 0.001), performance-approach goals (*β* = 0.04, *p* = 0.004), and performance-avoidance goals (*β* = −0.05, *p* = 0.001) contributed to autonomous writing motivation, via self-efficacy for regulation. Additionally, significant indirect paths were found between mastery goals (*β* = −0.04, *p* = 0.020), performance-approach goals (*β* = − 0.03, *p* = 0.039), performance-avoidance goals (*β* = 0.04, *p* = 0.020), and controlled writing motivation, via self-efficacy for regulation (*cf.*, *H2* + *H5*). Finally, mastery goals were positively related to writing performance, via autonomous writing motives (*β* = 0.06, *p* = 0.049) (*cf.*, *H4* + *H6*).

## 6. Discussion

In what follows, we elaborate on the direct and indirect relations found in the current path model. More particularly, building further on prior studies revealing the relations between implicit theories, achievement goals, and self-efficacy, we discuss the results related to hypotheses 1 and 2. Furthermore, extending prior studies relating motivational constructs to writing performance, we will zoom in on the role of writing motives in understanding this complex interplay (*cf.*, hypotheses 3 to 6). Throughout the discussion, we address the limitations of the study and offer directions for future research and we present the educational implications of this study for writing instruction.

### 6.1. Relating implicit theories, achievement goals, and self-efficacy

In line with the theoretical assumptions of ST and AGT (Elliot and Harackiewicz, 1996; Elliot and Church, 1997; Dweck, 1999) and based on prior writing research (Limpo and Alves, 2017; Camacho et al., 2022), the present results showed that students who believe that writing is a fixed and innate ability, have the tendency to avoid the appearance of incompetence in writing. In turn, these performance-avoidance goals undermine students’ self-efficacy for argumentation, regulation, and conventions (Soylu et al., 2017). Moreover, the current study also revealed an indirect negative relation between a fixed mindset regarding writing and students’ self-efficacy beliefs for writing, via performance-avoidance goals. Contrary to the negative role of performance-avoidance goals in predicting students’ self-efficacy, the results indicated that the more students are oriented to become better writers (i.e., mastery goals) or to maximize their perceived competence (i.e., performance-approach goals), the higher they perceive their ability to argument, to self-regulate during writing, and to adhere to writing conventions (Limpo and Alves, 2017; Soylu et al., 2017).

In sum, these findings highlight major educational implications, namely that it is key for students to be convinced that each and every student is able to learn to write provided that (1) they put in enough effort and time and (2) they are supported in this process. Herein lies a crucial role for today’s writing instruction and for teachers responsible for that instruction. That is, if students are not explicitly supported in learning when, what, and how to write, the vast majority of students will evidently fail in becoming good and effective writers (Graham et al., 2016) and unintendedly, effective writing may be perceived as a fixed trait that only a happy few are blessed with. To break through this misconception, high-quality writing instruction and explicit guidance is essential for students not only to become skillful writers, but also to help them experience and understand the development of writing from a growth mindset instead of a fixed mindset. In this respect, more experimental research is needed to understand how instructional practices can foster (groups of) students’ growth mindset or counter their fixed mindset regarding writing (e.g., Limpo and Alves, 2014; Camacho et al., 2023).

### 6.2. The role of writing motives

This study expands prior studies (Limpo and Alves, 2017; Soylu et al., 2017; Camacho et al., 2022) by including writing motives both as dependent variable (predicted by achievement goals and self-efficacy) and as independent variable (predicting students’ writing performance) in the path model. In discussing the role of writing motives, three key results are highlighted and discussed.

First, the results showed that students reporting higher levels of mastery goals were more driven by values or by inherent fulfillment of writing (i.e., autonomous writing motives), while students reporting higher levels of performance-approach or performance-avoidance goals were more driven by external or internal pressure to write (i.e., controlled writing motives). In this respect, the current study is the first to substantiate the alignment between AGT (Elliot and Harackiewicz, 1996; Elliot and Church, 1997) and SDT (Ryan and Deci, 2000b, 2020) in writing research by relating the trichotomous model of achievement goals with autonomous and controlled writing motivation. Further empirical research is needed in view of replication as well as in view of further in-depth investigation. Concerning the latter, we call for more qualitative research to get more fine-grained insights into students’ underlying goals and motives for writing (e.g., via interviews). In this way, we can learn (1) to understand how achievement goals and motives for writing are intertwined and (2) to uncover potential (instructional) factors hindering or facilitating students’ achievement goals and motives for writing.

Second, in line with prior research on students’ learning in general (Katz et al., 2014), the present results highlighted that the more students reported higher levels of self-efficacy for regulation, the more they were autonomously motivated to write and the less they were driven by controlled motives for writing. Moreover, the results also indicated that self-efficacy for regulation mediates the relation between achievement goals and writing motives. These results emphasize the key role of self-efficacy for regulation compared to the other two dimensions of self-efficacy for writing (i.e., self-efficacy for argumentation and for conventions). Although self-efficacy for regulation was not directly related to students’ writing performance in the current study (*cf.*, contrary to Limpo and Alves, 2017), its central position in the path model relating the different motivational concepts, warrants attention for further research. Given the relatively low mean score on self-efficacy for regulation (compared to the mean scores of self-efficacy for argumentation and for conventions) and given the evidence that self-efficacy for regulation is a key mechanism in understanding the relatedness of the motivational writing concepts, more experimental research is needed on how to foster students’ self-efficacy for regulation in particular. In this respect, a recent experimental study showed that providing secondary school students with explicit instruction regarding writing knowledge (i.e., text structure knowledge, genre knowledge) and writing strategies (e.g., planning, revising strategies) and enabling students to write in collaboration, fostered students’ self-efficacy for regulation (Landrieu et al., 2023). Next to replication studies on the effect of explicit writing instruction and collaborative writing on students’ self-efficacy for regulation, we call for more in-depth research to uncover how exactly students benefit from these instructional practices in terms of their self-efficacy for regulation (e.g., exploring which key ingredients of explicit writing instruction and collaborative writing are essential in nurturing students’ self-efficacy for regulation).

Finally, in line with theoretical SDT assumptions (Ryan and Deci, 2000b, 2020) and empirical evidence of prior studies (De Smedt et al., 2016, 2018b; Rasteiro and Limpo, 2022), students’ autonomous writing motivation was positively related to students’ argumentative writing performance. Furthermore, autonomous writing motivation mediated the positive relation between mastery goals and writing performance. These results highlight the importance of fostering students’ autonomous writing motivation in view of optimizing their writing performance. Herein lies a crucial role for today’s writing instruction: students do not only need to be taught writing skills, strategies, and knowledge to become skillful writers (Graham, 2018a,b). Being skillful in writing can help overcome the cognitive challenges writers face, but cannot overcome the motivational burdens of writing. To persevere in writing for different assignments with varying complexity, on different topics, using different genres, over longer periods of time with fluctuating levels of frustration, students need to be skillful *and* autonomously motivated writers. Being autonomously motivated refers to understanding the power and potential of writing for both authors and audience, recognizing the cognitive and motivational complexity involved, and being able to identify coping mechanisms to overcome these challenges. To support students in becoming autonomously motivated writers, today’s education needs to enable students to experience the value of writing in their educational, professional, and personal life or even to experience joy and pleasure when writing. According SDT, nurturing students’ inherent psychological need for autonomy, competence, and relatedness is key in fostering students’ autonomous motivation (Ryan and Deci, 2000a). In the context of writing education, teachers can adopt autonomy-supportive, structured, and involved teaching behavior by for example (a) providing students with choice of writing subjects, tools, or partners (*cf.*, the need for autonomy); (b) providing explicit instruction and clear writing goals so students know how to approach the writing assignment (*cf.*, need for competence), and (c) create a writing community in class in which students can share their writing and confer with each other on their writing process and product (*cf.*, need for relatedness). Experimental research on the effect of autonomy-supportive, structured, and involved teacher behavior on students autonomous writing motivation remains, however, extremely scarce (see De Smedt et al., 2018a) and is therefore strongly needed.

### 6.3. Limitations and suggestions for future research

In addition to the research suggestions already raised, we conclude with additional suggestions and acknowledge the limitations of the current study. First, although the path model revealed interesting relations between students’ implicit theories of writing, writing achievement goals, self-efficacy for writing, and writing motives, the proportion of variance in writing performance that can be explained by these motivational predictors remains small (2%). This potentially raises the question: if these motivational variables predict so little, what other factors should be considered to predict students’ writing performance? Next to student-level predictors such as individual background (e.g., students’ home language, socio-economic status) and cognitive factors (e.g., students’ applied writing strategies, basic writing skills), we especially want to stress the importance of class-level predictors such as instructional factors (e.g., instructional writing practices, amount of writing instruction, teacher expectations, teacher behavior) to predict students’ writing performance. In this respect, we call for future studies to include teacher or class-level variables in multilevel path models. Moreover, given the central role of instruction, we argue for more experimental research studying the effect of instructional writing practices on the interplay of the motivational predictors and students’ writing performance (e.g., multiple group path analyses to study significant differences in the paths between experimental and control conditions).

Second, the current study focused on writing performance in one genre, namely argumentative writing. Follow-up research should consider studying the interplay between these motivational variables and writing performance in different genres given that students’ motivation might differ depending the writing genre. More particularly, multiple-group path analyses can potentially reveal different paths between the motivational variables depending on the genre.

Finally, the present study used cross-sectional data to study the hypothesized relational model. We call for longitudinal research to study the mechanisms underlying the relations between the key motivational constructs and their role in predicting students’ writing performance. Longitudinal designs could also study how students’ writing performance, in turn, can affect students’ implicit theories of writing, writing achievement goals, self-efficacy for writing, and writing motivation.

## Data availability statement

The original contributions presented in the study are included in the article/supplementary material, further inquiries can be directed to the corresponding author.

## Ethics statement

The studies involving human participants were reviewed and approved by The Faculty of Psychology and Educational Sciences of Ghent University (Specific Ethical Protocol for Scientific Research). Written informed consent for participation was not provided by the participants’ legal guardians/next of kin because: There was an active written informed consent from the participants and a passive written informed consent from participants’ parents.

## Author contributions

FDS was in charge of the design of the study, data analysis, and she wrote the manuscript. YL was in charge of data collection. All authors contributed to the manuscript and approved the submitted version.

## Funding

This research was supported by the Research Foundation Flanders (FWO) under Grant G010719N.

## Conflict of interest

The authors declare that the research was conducted in the absence of any commercial or financial relationships that could be construed as a potential conflict of interest.

## Publisher’s note

All claims expressed in this article are solely those of the authors and do not necessarily represent those of their affiliated organizations, or those of the publisher, the editors and the reviewers. Any product that may be evaluated in this article, or claim that may be made by its manufacturer, is not guaranteed or endorsed by the publisher.

## Footnotes

^1^We evaluated an alternative model which we compared with the current hypothesized model (see Figure 1). More particularly, in this alternative model we included a direct path between implicit theories and writing performance on the one hand, and between achievement goals and writing performance on the other hand. Although this alternative model fitted the data as well as the current hypothesized model (*χ*^2^ (6) = 23.49, *p* < 0.001, CFI = 0.977, RMSEA = 0.087, SRMR = 0.040), none of the added paths were statistically significant (implicit theories and writing performance: *β* = 0.05, *p* = 0.37; mastery goals and writing performance: *β* = 0.09, *p* = 0.17; performance-approach goals and writing performance: *β* = 0.00, *p* = 0.98; and performance-avoidance goals and writing performance: *β* = −0.13, *p* = 0.06). These results suggest acceptance of the current hypothesized model as the most parsimonious.
